# Mini-Screw-Assisted Orthodontic Retraction of Class I Malocclusion in a Bimaxillary Protrusion Patient Using Sliding Mechanics: A Case Report

**DOI:** 10.7759/cureus.57665

**Published:** 2024-04-05

**Authors:** Rozina Vishnani, Rizwan Gilani, Shefali Singh, Shruti Rathi, Anjali Kathade

**Affiliations:** 1 Oral and Maxillofacial Surgery, Sharad Pawar Dental College and Hospital, Datta Meghe Institute of Higher Education & Research, Wardha, IND; 2 Orthodontics and Dentofacial Orthopaedics, Sharad Pawar Dental College and Hospital, Datta Meghe Institute of Higher Education & Research, Wardha, IND

**Keywords:** absolute anchorage, bimaxillary protrusion, tads, mini-screw, retraction

## Abstract

A condition known as bimaxillary protrusion occurs when the front teeth protrude due to the forward positioning of the lower and upper jaws. Temporary anchorage devices (TADs) are utilized to provide anchorage and facilitate the controlled retraction of maxillary and mandibular protruding teeth, helping to correct the patient’s bite and facial aesthetics. A 27-year-old female with bimaxillary protrusion reported to the Department of Orthodontics. On examination, the facial profile of the patient was convex. The clinical FMA was high. With a deep mentolabial sulcus and an acute nasolabial angle, lips were potentially competent. An intraoral examination revealed proclined incisors with spacing in the maxillary arch and proclined anterior teeth in the mandibular arch. Space closure was done using sliding mechanics with direct anchorage from a mini-screw after the extraction of all four first premolars. There was a significant improvement in the patient’s profile posttreatment.

## Introduction

Bimaxillary protrusion, also known as protrusive maxillary dentoalveolar dysplasia or protrusive maxilla-mandibular dentoalveolar dysplasia, is a condition characterized by the forward positioning of both the upper (maxilla) and lower (mandible) jaws and teeth. This results in an exaggerated protrusion of the lips and an outward appearance of the face [1].

Mini-screw-assisted retraction in Class I bimaxillary (both upper and lower jaws) patients using sliding mechanics is a common orthodontic treatment approach. With this method, the anterior teeth are moved backward in order to treat a variety of orthodontic problems, including crowding, spacing, and other malocclusions [2]. Mini-screw retraction is a successful orthodontic treatment method for patients with bimaxillary protrusion. Bimaxillary protrusion is a condition where both the upper and lower jaws are positioned forward, causing the front teeth to protrude [3]. Temporary anchorage devices (TADs) are utilized to provide anchorage, facilitate the controlled retraction of anterior protruding teeth, and improve facial aesthetic and functional occlusion [4].

The use of TADs in patients with bimaxillary protrusion allows for more predictable and controlled tooth movement while minimizing the risk of anchor loss. It can significantly improve the look and functionality of the patient’s smile. Treatment plans may vary depending on the specific patient’s needs and the orthodontist’s expertise. A professional orthodontist should be consulted for a customized evaluation and treatment plan [5].

It is important to remember that the particular treatment plan may change based on the orthodontist’s preferred methods and the particular instance of the patient. Mini-screw-assisted retraction with sliding mechanics has become a valuable tool in orthodontics, allowing for more efficient and predictable tooth movement, especially in complex cases like Class I bimaxillary patients with significant anterior crowding or protrusion [6].

## Case presentation

**Figure 1:**
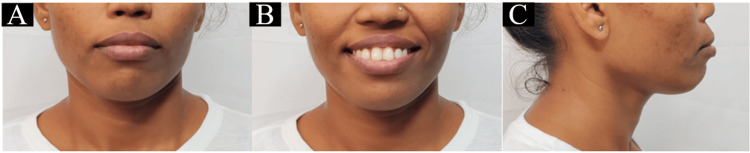
Pretreatment extraoral photographs: (a) frontal, (b) smiling, and (c) profile

A 27-year-old female patient reported to the Department of Orthodontics and Dentofacial Orthopedics, with the primary concern of forwardly placed upper front teeth. On extraoral examination, mesoprosopic facial morphology was seen. The patient exhibited a convex facial profile on profile assessment. The clinical FMA was high. Lips were potentially competent with an acute nasolabial angle and deep mentolabial sulcus (Figure 1).

**Figure 2:**
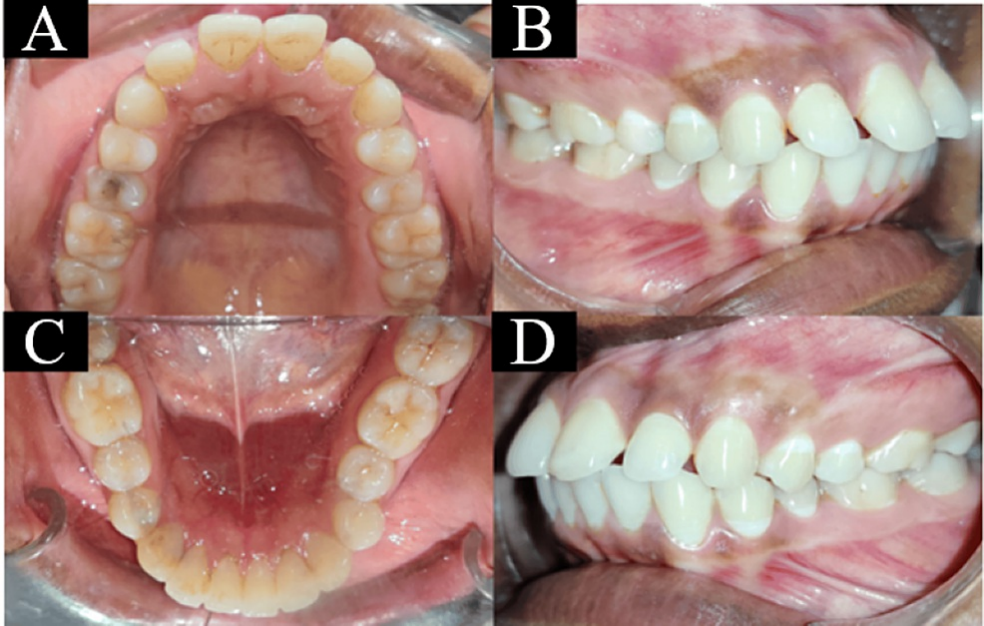
Pretreatment intraoral photographs: (a) maxillary arch, (b) right occlusion, (c) mandibular arch, and (d) left occlusion

On intraoral examination, proclined incisors with spacing in the maxillary arch and proclined anterior teeth in the mandibular arch were seen. Both molars and canines were in Angle’s Class I malocclusion (Figure 2).

**Figure 3:**
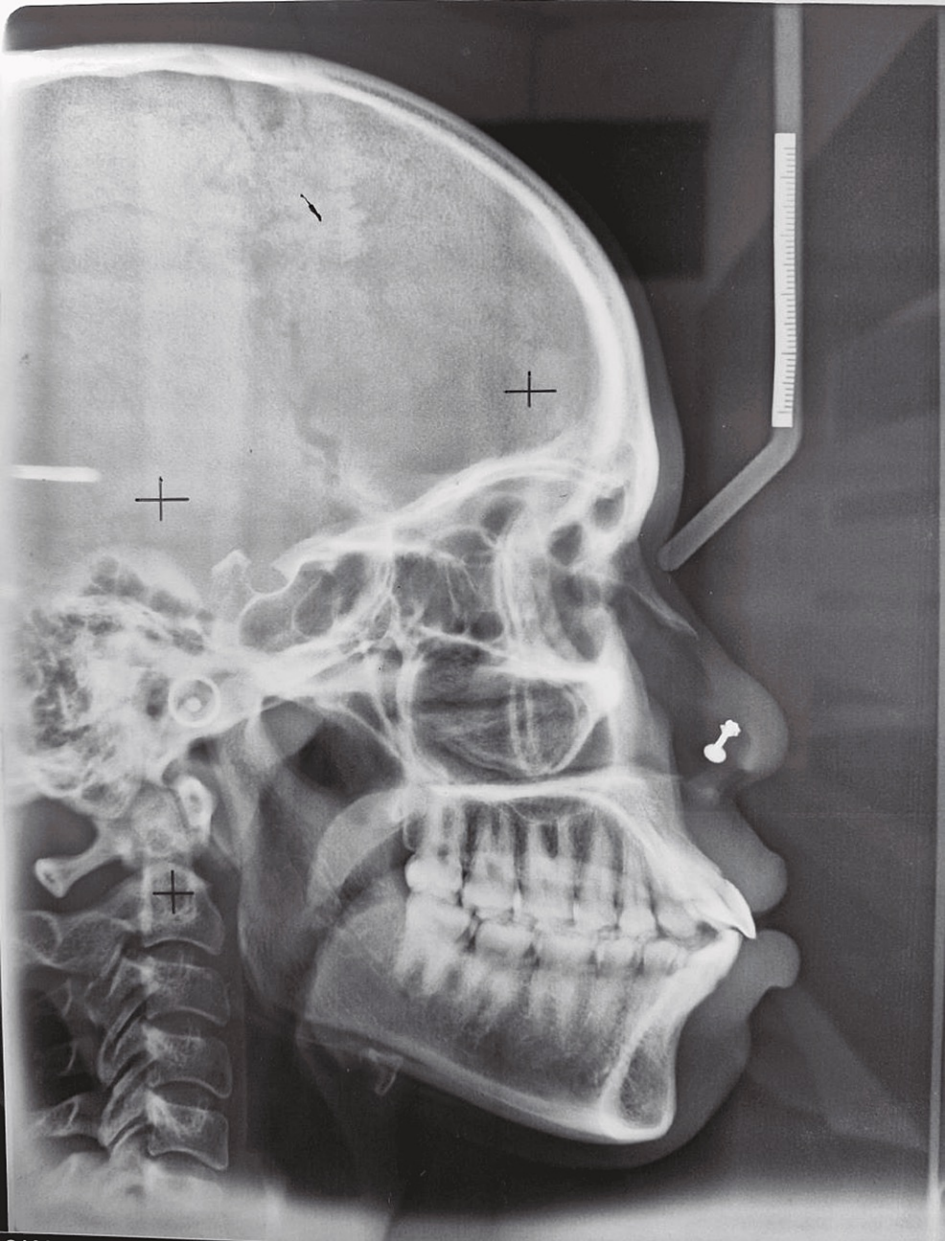
Pretreatment lateral cephalogram

On cephalometric examination, the patient had 1 to NA of 28 degrees and 1 to NB of 43 degrees, Class I skeletal bases (ANB angle was 2 degrees and Beta angle was 41 degrees), a horizontal growth pattern (FMPA angle was 24 degrees), and was at CVMI stage IV (complete), according to cephalometric analysis (Figure 3).

**Figure 4:**
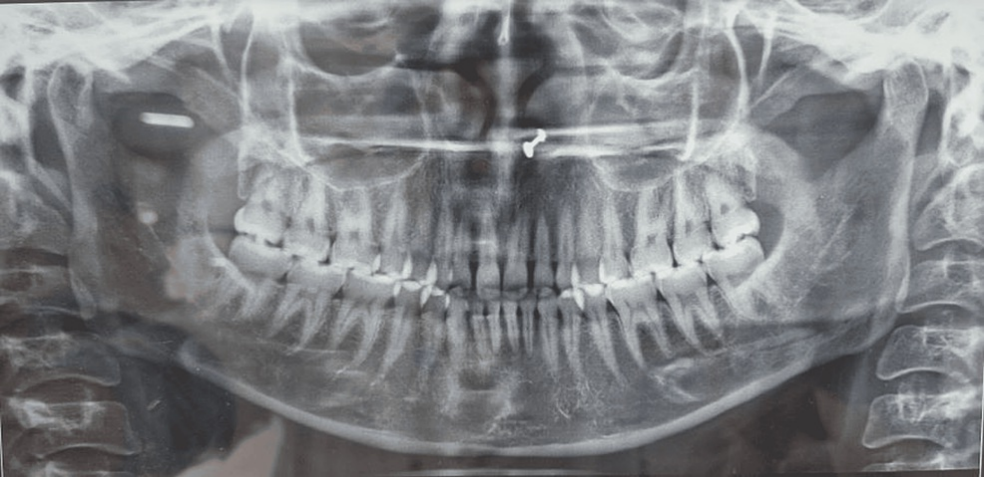
Pretreatment OPG OPG, orthopantomography

The pretreatment orthopantomography (OPG) revealed all teeth present in all four quadrants with an adequate amount of bone support and no bone loss (Figure 4).

The treatment options were retraction and closure of the extraction space by sliding mechanics using the elastomeric chain or by the placement of TADS. Treatment objectives were to align the maxillary and mandibular arch, achieve adequate overjet and overbite, closure of spaces, and reduction of proclination of anterior teeth while maintaining Class I molar and Class I canine relations on both sides.

Orthodontic phase

**Figure 5:**
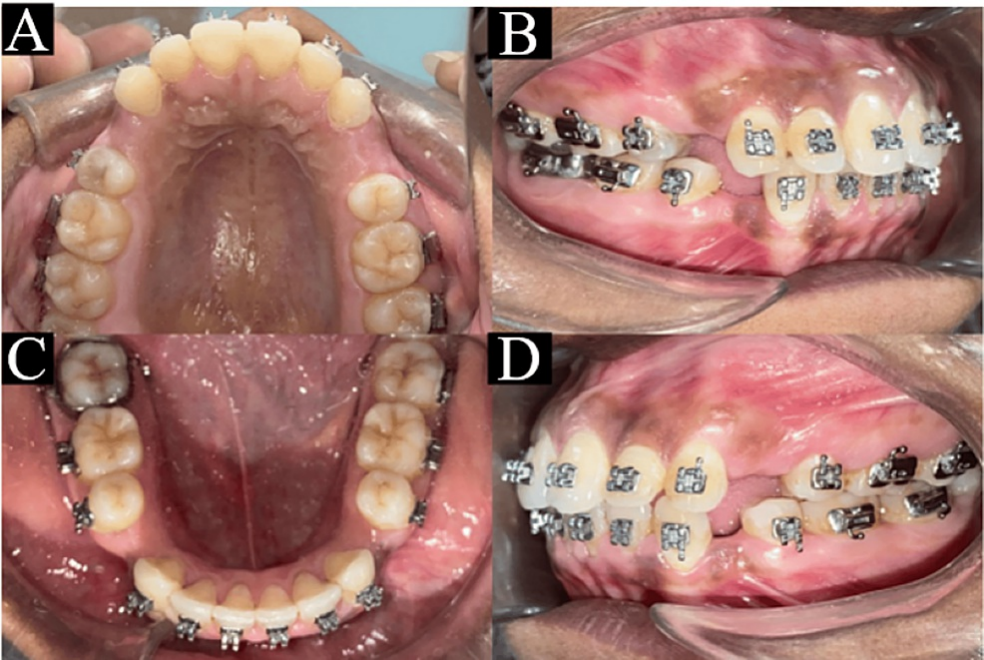
Mid-treatment intraoral photographs: (a) maxillary arch, (b) right occlusion, (c) mandibular arch, and (d) left occlusion

Treatment was started by strapping up using an MBT 0.022” slot bracket system. Initial leveling and alignment of the arches with 0.019 × 0.025 stainless steel wire were done. After the closure of all the interdental spaces and achieving well-aligned arches, the extraction of the first premolars from all arches was done (Figure 5).

**Figure 6:**
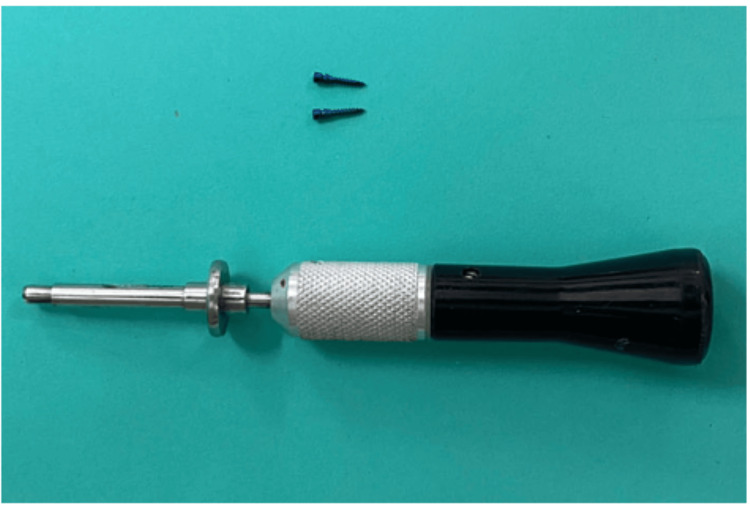
TAD driver and mini-screws TAD, temporary anchorage device

Mini-screws of dimension 1.5 × 8 mm were placed on the buccal side between first molars and second premolars at the level of 5-6 mm apical to alveolar crest in all four quadrants using TAD driver (Figure 6).

**Figure 7:**
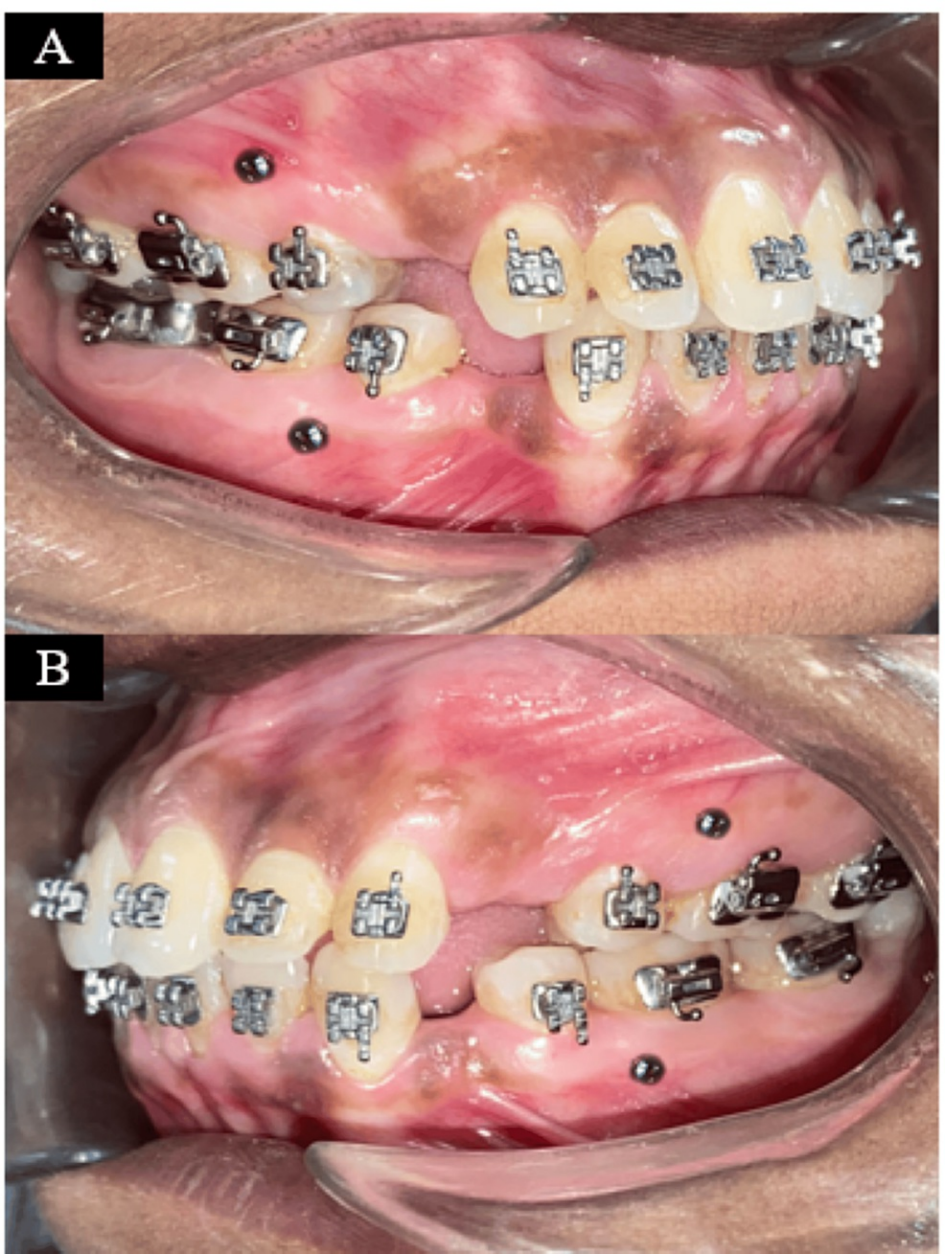
Placement of TADs: (a) right occlusion and (b) left occlusion TADs, temporary anchorage devices

Space closure was done using sliding mechanics with direct anchorage from the mini-screw, as they can achieve maximum anchorage en masse retraction (Figure 7). It provided absolute anchorage while retraction of maxillary and mandibular anterior segments.

**Figure 8:**
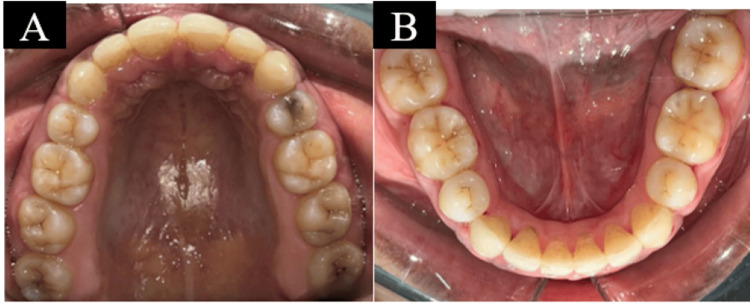
Retraction and space closure: (a) maxillary arch and (b) mandibular arch

The following goals were accomplished following the retraction: alignment of the mandibular and maxillary anterior teeth, space closure in the upper anterior teeth, and retraction and space closure with TADs without anchorage loss, as shown in Figure 8.

**Figure 9:**
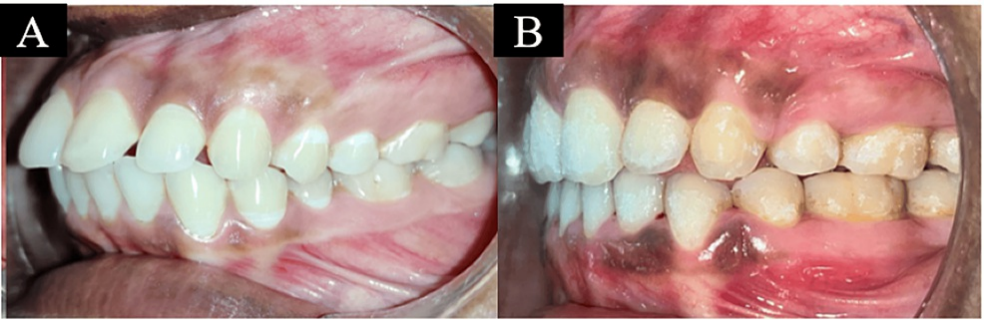
Reduction of overjet: (a) pretreatment left occlusion and (b) posttreatment left occlusion

After complete retraction and space closure, a normal overjet and overbite were achieved when compared to the pretreatment photograph, as shown in Figure 9.

**Figure 10:**
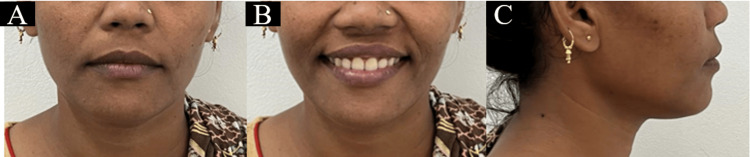
Posttreatment extraoral photographs: (a) frontal, (b) smiling, and (c) profile

Posttreatment extraoral photographs depict a significant reduction in the convexity of the face, achievement of lip competency, and improved smile of the patient (Figure 10).

**Figure 11:**
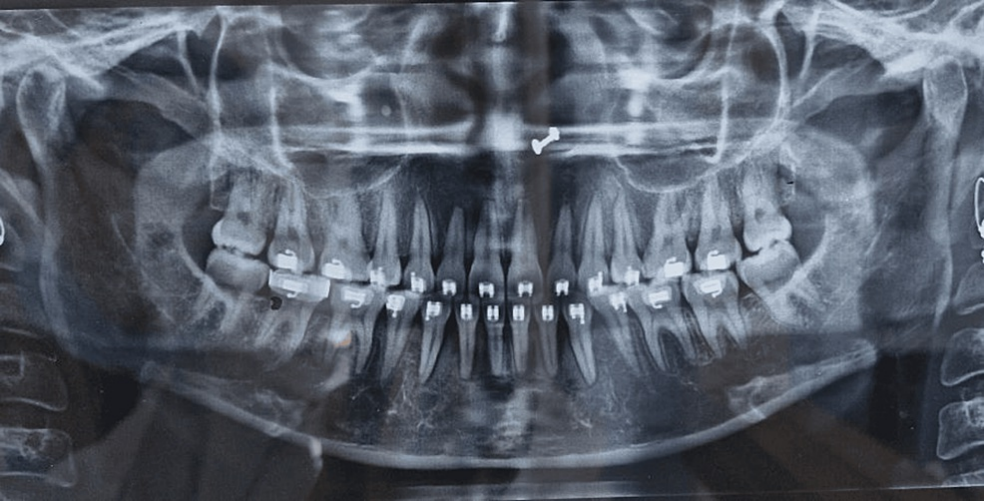
Posttreatment OPG OPG, orthopantomography

OPG taken during the finishing and detailing stage revealed parallelism of roots, as shown in Figure 11.

**Figure 12:**
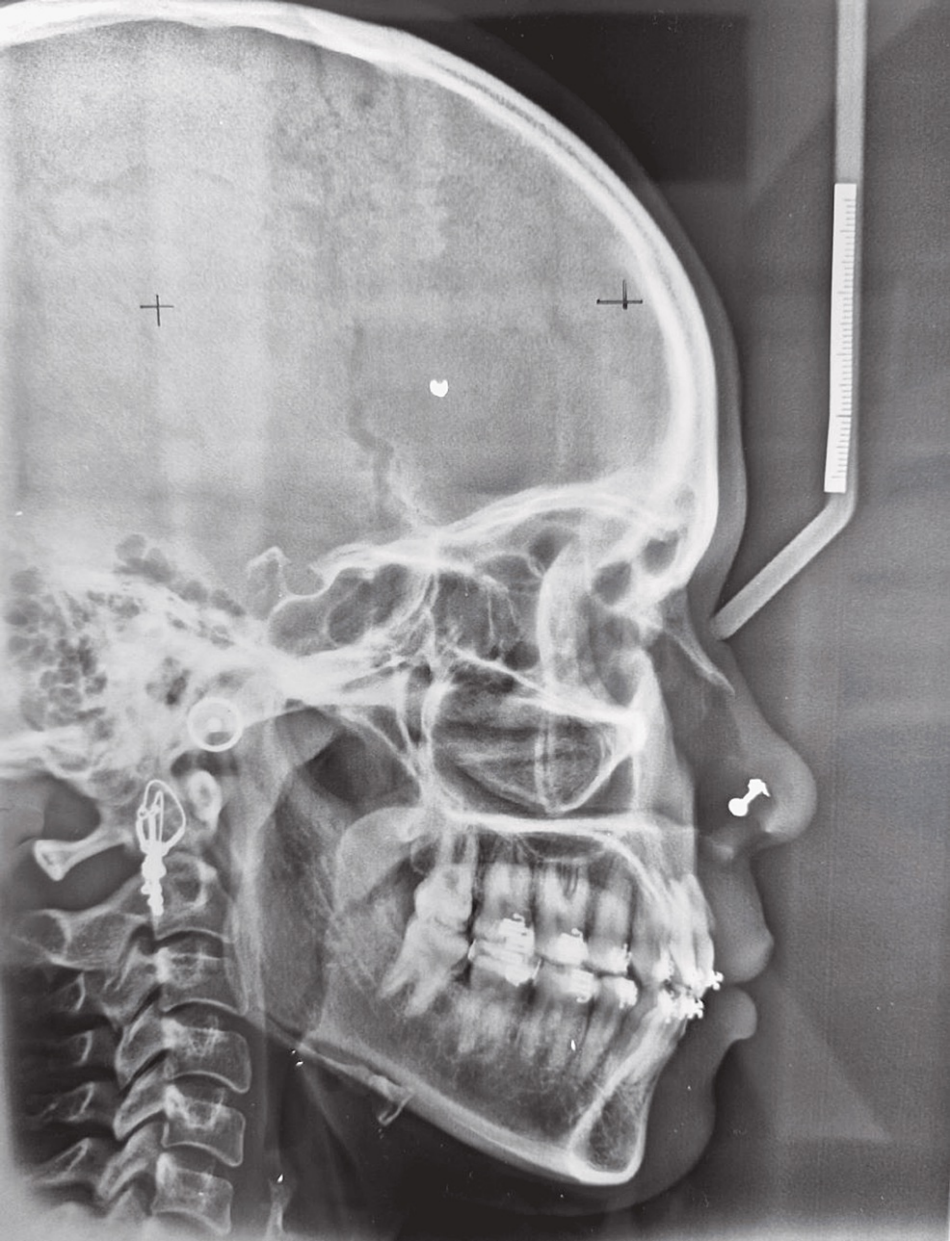
Posttreatment lateral cephalogram

The posttreatment cephalogram showed a significant reduction in the proclination of both upper and lower anterior teeth without anchorage loss while maintaining Class I molar and canine relationship (Figure 12).

## Discussion

The treatment of Class I bimaxillary protrusion, a condition where both the upper and lower teeth are forwardly positioned within a normal skeletal relationship, typically involves orthodontic and sometimes orthognathic (jaw) treatment. The primary goal is to correct the dental and skeletal discrepancies to achieve a balanced and harmonious facial profile. Depending on the severity of the protrusion and crowding, orthodontists may recommend extracting one or more teeth to create space for the retraction of protruding teeth [7].

It is important for orthodontists to carefully plan retraction and space closure in bimaxillary protrusion cases to achieve optimal outcomes while minimizing potential anchor loss. Over-retraction of anterior teeth can lead to a flattened facial profile and compromised lip support, which may negatively impact the patient’s facial aesthetics. Additionally, inadequate space closure can result in residual gaps or relapse of the protrusion posttreatment [8].

The timing and sequence of retraction and space closure are also crucial considerations in treatment planning. Sequential retraction of the anterior teeth followed by space closure in the posterior segments can help distribute forces evenly and minimize the risk of undesirable tooth movements [9].

Biomechanical systems are designed to apply forces that produce controlled tooth movement. In bimaxillary protrusion cases, retraction forces are typically directed in a posterior and downward direction to retract the protruded teeth into the dental arch. The use of TADs allows for the application of these forces without relying on anchorage from neighboring teeth [10].

TADs have revolutionized orthodontic treatment by providing reliable and stable anchorage. In Class I bimaxillary protrusion cases, where controlling anchorage is crucial, TADs play a pivotal role in preventing undesired tooth movement during retraction. The case report highlights the strategic placement of TADs to enhance biomechanics and ensure efficient retraction without compromising the stability of the results [11].

Beyond clinical efficacy, TAD-supported retraction in Class I bimaxillary protrusion cases enhances the overall patient experience. Reduced treatment duration, fewer appointments, and minimized discomfort contribute to improved patient satisfaction [12].

## Conclusions

For additional anchorage during orthodontic treatment, mini-screws or TADs might be utilized. When retracting anterior teeth, in particular, they provide exact control over tooth movement. As orthodontic practices evolve, TADs continue to shape innovative solutions, providing orthodontists with a valuable tool to achieve optimal outcomes in challenging cases.
